# Three-dimensional ultrashort echo time magnetic resonance imaging in pediatric patients with pneumonia: a comparative study

**DOI:** 10.1186/s12880-023-01130-2

**Published:** 2023-11-02

**Authors:** Yan Sun, Yujie Chen, Xuesheng Li, Yi Liao, Xijian Chen, Yu Song, Xinyue Liang, Yongming Dai, Dapeng Chen, Gang Ning

**Affiliations:** 1 Department of Radiology, West China Second Hospital, Sichuan University, No.1416, Section 1, Chenglong Road, Chengdu, Sichuan Province 610066 People’s Republic of China; 2 Key Laboratory of Birth Defects and Related Diseases of Women and Children (Sichuan University), Ministry of Education, Chengdu, Sichuan Province 610041 People’s Republic of China; 3 Central Research Institute, United Imaging Healthcare, Shanghai, People’s Republic of China; 4 Department of Pediatrics, West China Second Hospital, Sichuan University, No.1416, Section 1, Chenglong Road, Chengdu, Sichuan Province 610066 People’s Republic of China

**Keywords:** Pediatric pneumonia, Neonates, Lung, Ultrashort echo time, Magnetic resonance imaging

## Abstract

**Background:**

UTE has been used to depict lung parenchyma. However, the insufficient discussion of its performance in pediatric pneumonia compared with conventional sequences is a gap in the existing literature. The objective of this study was to compare the diagnostic value of 3D-UTE with that of 3D T1-GRE and T2-FSE sequences in young children diagnosed with pneumonia.

**Methods:**

Seventy-seven eligible pediatric patients diagnosed with pneumonia at our hospital, ranging in age from one day to thirty-five months, were enrolled in this study from March 2021 to August 2021. All patients underwent imaging using a 3 T pediatric MR scanner, which included three sequences: 3D-UTE, 3D-T1 GRE, and T2-FSE. Subjective analyses were performed by two experienced pediatric radiologists based on a 5-point scale according to six pathological findings (patchy shadows/ground-glass opacity (GGO), consolidation, nodule, bulla/cyst, linear opacity, and pleural effusion/thickening). Additionally, they assessed image quality, including the presence of artifacts, and evaluated the lung parenchyma. Interrater agreement was assessed using intraclass correlation coefficients (ICCs). Differences among the three sequences were evaluated using the Wilcoxon signed-rank test.

**Results:**

The visualization of pathologies in most parameters (patchy shadows/GGO, consolidation, nodule, and bulla/cyst) was superior with UTE compared to T2-FSE and T1 GRE. The visualization scores for linear opacity were similar between UTE and T2-FSE, and both were better than T1-GRE. In the case of pleural effusion/thickening, T2-FSE outperformed the other sequences. However, statistically significant differences between UTE and other sequences were only observed for patchy shadows/GGO and consolidation. The overall image quality was superior or at least comparable with UTE compared to T2-FSE and T1-GRE. Interobserver agreements for all visual assessments were significant and rated “substantial” or “excellent.”

**Conclusions:**

In conclusion, UTE MRI is a useful and promising method for evaluating pediatric pneumonia, as it provided better or similar visualization of most imaging findings compared with T2-FSE and T1-GRE. We suggest that the UTE MRI is well-suited for pediatric population, especially in younger children with pneumonia who require longitudinal and repeated imaging for clinical care or research and are susceptible to ionizing radiation.

## Background

Pneumonia, a condition caused by infection of the lower respiratory tract and lung parenchyma, frequently affects the pediatric population [1]. It is the leading cause of mortality in young individuals and a condition associated with a significant disease burden [2–5]. The manifestations of pediatric pneumonia are diverse and variable, and imaging assessments of the pulmonary structure play a crucial role in its diagnosis and treatment. Although chest radiography is commonly used as the initial radiologic investigation [6, 7], it has substantial interobserver variability of interpretation [8, 9] and limitations in depicting the extent of pneumonia and its complications [10]. As a result, CT has been considered the preferred imaging modality [11, 12]. However, CT involves more exposure to ionizing radiation [10], which is not suitable for the longitudinal evaluation of pediatric patients [13, 14]. To refrain from exposure to ionizing radiation, MRI has emerged as an attractive alternative with superior soft-tissue contrast and favorable spatial resolution [12, 15]. The conventional T1- and T2-weighted sequences have demonstrated diagnostic value in pediatric patients with pneumonia [16]; however, these traditional sequences have limitations in depicting the radiographic findings of parenchymal nodules [17] and air-trapping lesions [18] compared to CT. Due to the low proton density of the lungs and the very rapid transverse relaxation rate [12], conventional spin-echo or gradient-echo sequences with longer echo times struggle to detect lung signals [11].

With improvements in MRI, ultrashort echo time (UTE) imaging techniques satisfactorily depict lung parenchyma [19] and have been considered as “game changers” in lung MRI by Fleischner Society [20, 21]. UTE shows promise in patients with cystic fibrosis, hyperinflation, emphysema pneumopathies, and infection [18], providing better visualization of intrinsic MRI signals of the lung. It demonstrates performance equivalent to CT in detecting bronchial alterations and superior capacity in depicting parenchymal changes compared to conventional T1 and T2 sequences [20]. The characteristic bronchial or parenchymal performance often indicates specific diseases and may influence therapeutic strategy in clinical practice. For instance, the presence of necrosis/abscesses often indicated longer antibiotic treatments and potential interventional procedures [16]. Accordingly, the more detailed MRI sequences provided in the evaluation of the lung infection, the better for diagnoses and treatments, especially in the vulnerable pediatric population.

Although previous studies have compared UTE and T2-weighted image (T2WI) sequences in childhood with pneumonia [18], the T1-weighted image (T1WI) sequence was not included, and the value of UTE to younger children, especially neonates, has not been specifically addressed. To date, a universally accepted MRI technique for pediatric pneumonia has barely been established [10], and the diagnostic value of UTE in pediatric pneumonia, particularly in neonates, compared with conventional T1 and T2 sequences, remains insufficiently discussed.

This study aimed to explore the clinical value of the UTE sequence in young children with pneumonia, particularly newborns, and investigate a radiation-free imaging approach with diagnostic advantages. This study fills a gap in previous research and focuses on the assessment of the diagnostic superiority of three‑dimensional UTE in comparison with the standard routine sequences of T1-Gradient Echo (T1-GRE) and T2-Fast-Spin Echo (T2-FSE) in pulmonary pathological findings of pneumonia in children aged 0–3 years.

## Methods

### Study design and patients

Pediatric patients duly diagnosed with pediatric pneumonia by board-certified pediatricians per their laboratory examination results and clinical presentations such as fever, shortness of breath, coughing, expectoration etc. at our hospital were enrolled in this study from March 2021 to August 2021. Our inclusion criteria were as follows: (i) age below 5 years; (ii) no contraindication to MRI (ferromagnetic implants, pacemaker, etc.); (iii) informed consent of legal guardians given and voluntary participation in the study; (iv) children in stable conditions and capable of cooperating when performing MRI examinations. Exclusion criteria were as follows: (i) termination of MRI midway due to an unexpected accident/incident (such as unstable vital signs of patients); (ii) lack of clinical information; (iii) the radiological sign of pulmonary infection could not readily be evaluated.

Seventy-seven children were enrolled in this study, including 19 neonates and 58 non-neonates. The ages of our participants ranged from one day to thirty-five months. Among them, there were 33 born prematurely and 44 were birth at term. Nine of the 33 born prematurely had a history of bronchopulmonary dysplasia (BPD). Fifty of 77 subjects used the ventilator at birth during hospitalization (Table 1).

**Table 1:** Clinical characteristics of patients

Clinical characteristics		
	Range	Mean ± SD
Age at MRI (months)	0.03–35.73	5.75 ± 5.37
PMA (months)	7.93–44.13	14.22 ± 5.35
Gestational age (weeks)	25.71–41.71	36.29 ± 3.91
Birth weight (g)	750–4500	2692.51 ± 860.36
Gender	Male/Female	49/28
Ventilator at birth	Cases (rate)	50 (64.9%)
BPD	Cases (rate)	9 (11.7%)

*PMA* postmenstrual age, *BPD* bronchopulmonary dysplasia

All children were examined under sedation with MR-compatible anesthesia machines and monitors (MAGLIFE C PLUS and Fabius MRI) throughout the process. The administration of anesthesia was performed by skilled anesthesiologists, utilizing the inhalational anesthetic agent sevoflurane. Each pediatric patient received a pre-anesthetic assessment and post-anesthetic recovery under the supervision of the anesthesiologist. The legal guardians of children provided informed consent for the anesthesia procedure.

### MRI protocol

MR imaging was performed using a 3-Tesla (3 T) pediatric MR system (Alpha, United Imaging Healthcare, Shanghai, China). All examinations were performed under free breath, and an 8‐channel chest coil was employed. MRI was performed with the patients in a supine position. When the respiration was shallow and fast, the navigation gating acquisition mode was employed, and when it was deep and regular, we switched to respiratory gating mode. The three‑dimensional Ultrashort Echo time (3-D UTE) sequences acquired in the axial, coronal, and sagittal planes were reconstructed. The 3D T1-GRE and T2-FSE were both acquired in the axial plane. These parameters are shown in Table 2.

**Table 2:** Technical parameters of the MRI sequences

Parameters	UTE	T1-GRE	T2-FSE
TR in ms	1.7	3.58	2016
TE in ms	0.1	1.7	105
Flip angle in °	3	10	100
Acquisition matrix	192	192	192
Field of view in mm	200	200	200
Slice orientation	Axial	Axial	Axial
Breath-hold	No	No	No
Acquisition time in min	1–2	0.5–1.5	1–1.5

*TR* repetition time, *TE* echo time

### Imaging evaluation

Two pediatric radiologists with more than five years of work experience performed image evaluations on consensus. Both radiologists were blinded to all clinical information.

In the first step, image quality of visualization of the lung, including the presence of artifacts and lung parenchyma, was assessed by the two radiologists working separately. The extent of artifacts was scored on a four-point scale from 4 to 1 as follows: severe artifacts (4, nondiagnostic), moderate artifacts (3, limited diagnosis), mild artifacts (2, little or no effect on diagnosis), no artifacts (1). To assess the overall quality of the lung parenchyma, the following four-point scale was adopted: 1, with no signal intensity (indistinguishable from air); 2, fair image quality, with minimal signal intensity (barely distinguishable from air); 3, good image quality (clearly distinguishable from air but lung fissures were not visible); 4, excellent image quality (lung fissures were visible).

In the second step, all three sequences were compared. The images were interpreted with respect to the six pathological imaging findings, including patchy increased intensity, atelectasis/consolidation, nodule/mass, bulla/cyst, interstitial lung disease/pulmonary fibrosis, and pleural effusion/thickening in all three sequences. A five-point visual scale (0, absent; 1, equivocal; 2, poorly delineable; 3, well delineable; 4, excellently delineable) was adopted to assess the diagnostic performance of the three sequences. When there was nonconformity in the classification of pathological lesions, a senior radiologist with over 30 years of relevant experience adjudicated.

### Statistical analysis

The statistical analysis was carried out in IBM® SPSS® Statistics 25. The value of the intraclass correlation coefficient (ICC) was calculated to evaluate the interobserver agreement. The paired Wilcoxon signed-rank test was used to compare all the visual scores between the three sequences, including UTE, 3D-GRE, and T2-FSE. A *p*-value of less than 0.05 was considered statistically significant.

## Results

### Population characteristics

**Fig. 1:**
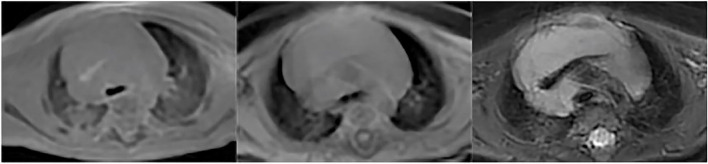
Patchy increased intensity. Patchy increased intensity in a 3-month-old boy with pneumonia. UTE, T1WI and T2WI were shown above in sequence. UTE sequence better delineate the patchy increased intensity than T2-FSE and 3D-T1 GRE

**Fig. 2:**
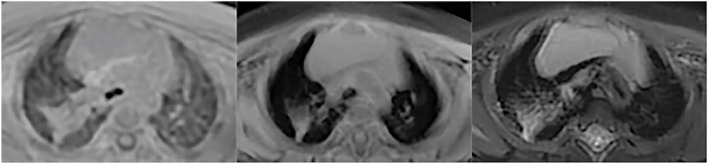
Atelectasis/consolidation. Consolidation in a 4-month-old boy with pneumonia. UTE, T1WI and T2WI were shown above in sequence. UTE sequence better delineate the consolidation than T2-FSE and 3D-T1 GRE

**Fig. 3:**
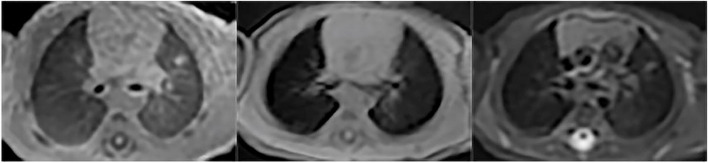
Nodule/mass. Nodule in a 16-day-old boy with pneumonia. UTE, T1WI and T2WI were shown above in sequence. UTE sequence better delineate the nodule than T2-FSE and 3D-T1 GRE

**Fig. 4:**
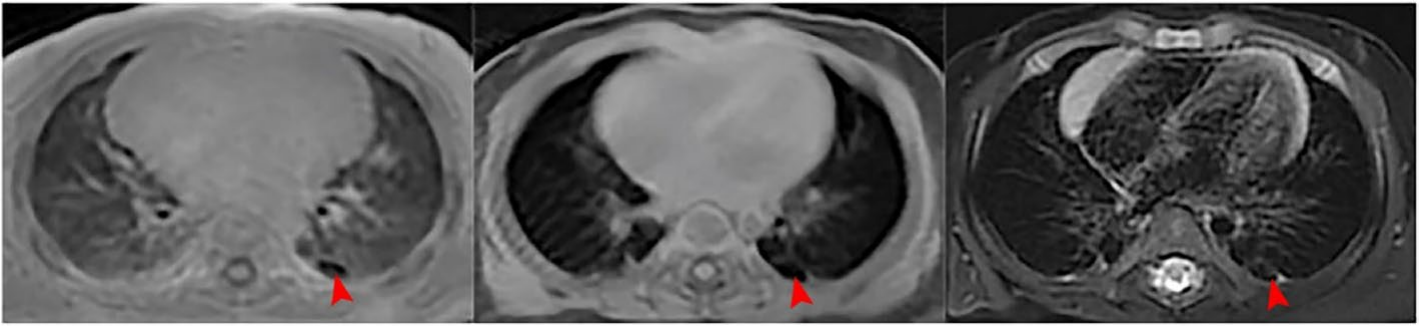
Bulla/cyst. Bullae in a 5-month-old boy with cough. UTE, T1WI and T2WI were shown above in sequence. UTE sequence better delineate the bullae (Red arrow) than T2-FSE and 3D-T1 GRE

**Fig. 5:**
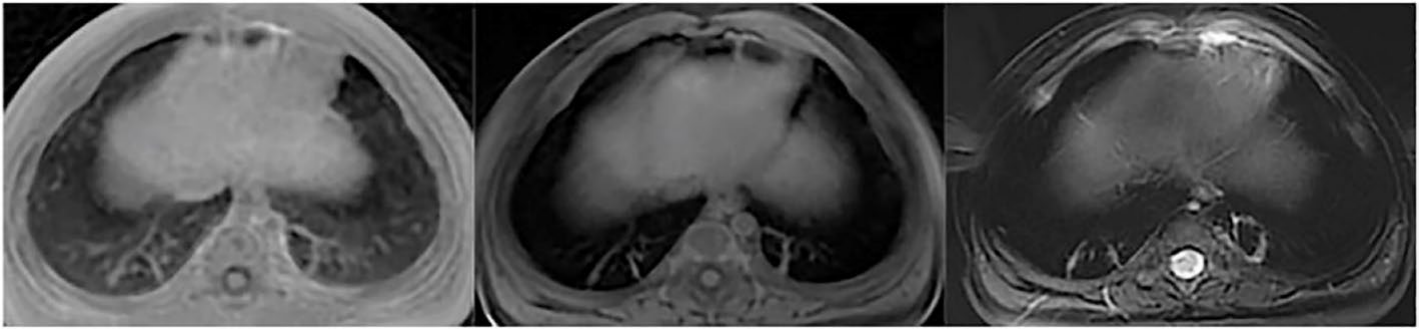
Interstitial lung disease/pulmonary fibrosis. Pulmonary fibrosis in a 5-month-old boy with pneumonia. UTE, T1WI and T2WI were shown above in sequence. UTE sequence better delineate the pulmonary fibrosis than 3D-T1 GRE, and comparable

**Fig. 6:**
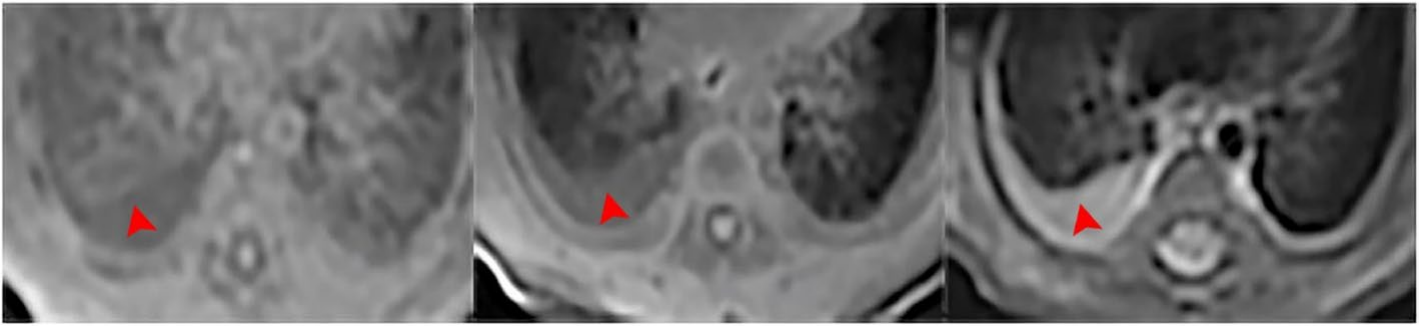
Pleural effusion/ thickening. Pleural effusion in a 18-day-old boy with neonatal pneumonia. UTE, T1WI and T2WI were shown above in sequence. T2-FSE was preferably depicted the pleural effusion (Red arrowhead) with favorable contrast than 3D-T1 GRE and UTE

Six pathological imaging findings were evaluated: patchy increased intensity (Fig. 1) was found in 69 children, atelectasis/consolidation (Fig. 2) in 15 children, nodule/mass in 3 children (Fig. 3), bulla/cyst in 4 children (Fig. 4), interstitial lung disease/pulmonary fibrosis in 26 children (Fig. 5), and pleural effusion/ thickening in 4 children (Fig. 6).

### Image evaluation

#### Overall image quality

##### Artifacts

All interobserver agreements for all sequences were significant and found to be “excellent” (UTE: ICC value = 0.86; *p* < 0.0001; T2-FSE: ICC value = 0.98; *p* < 0.0001; T1-GRE: ICC value = 0.89; *p* < 0.0001).

**Fig. 7:**
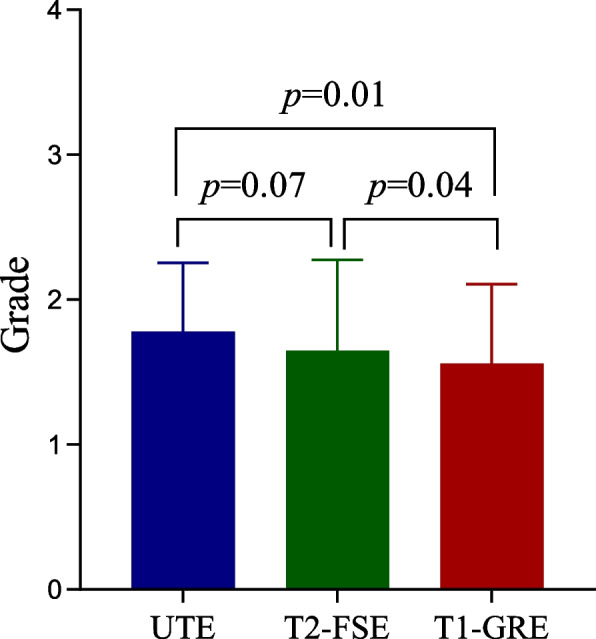
Comparison of image quality ratings for the artifacts. The image artifacts were slightly higher for TUE and T2-FSE than for T1-GRE, while there was no significant difference between TUE and T2-FSE

None of the examinations were rated as nondiagnostic with regard to artifacts. All three images of one patient were defined as limited diagnoses. The image artifacts that may interfere with the evaluation of imaging findings of pneumonia were slightly higher for TUE (Mean ± SD, 1.78 ± 0.48) and T2-FSE (1.65 ± 0.62) than for T1-GRE (1.56 ± 0.55) (*p* = 0.01 and 0.04, respectively), while there was no significant difference between TUE and T2-FSE (*p* = 0.07). A comparison of image quality ratings for the artifacts is shown in Fig. 7.

##### Lung parenchyma

On pulmonary parenchyma depiction, all interobserver agreements on each sequence were significant and found to be “excellent” (UTE: ICC value = 0.94; *p* < 0.0001; T2-FSE: ICC value = 0.84; *p* < 0.0001; T1-GRE: ICC value = 0.87; *p* < 0.0001).

**Fig. 8:**
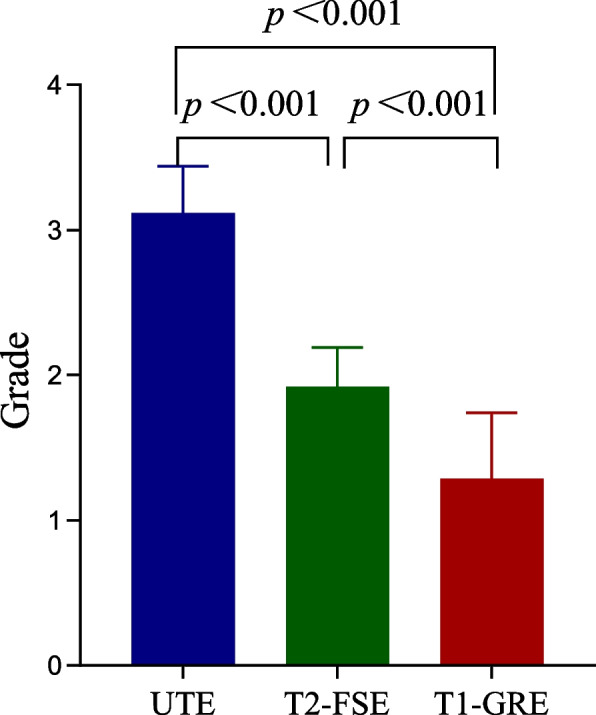
Comparison of visibility of lung parenchyma. The image quality of the lung parenchyma on UTE was significantly better than T2-FSE and T1-GRE, and the lung parenchyma was imaged better in T2-FSE than in T1-GRE

The image quality of the lung parenchyma was significantly better for UTE (3.12 ± 0.32) than for T2-FSE (1.92 ± 0.27) (*p* < 0.001) and T1-GRE (1.29 ± 0.45) (*p* < 0.001), and the lung parenchyma was imaged better in T2-FSE than in T1-GRE (*p* < 0.001). Figure 8 shows a comparison of the visibility of the lung parenchyma.

#### Radiological findings

Interobserver agreements for the assessment of all imaging findings on each sequence were significant and found to be “substantial” or “excellent,” with the ICC value ranging from 0.75 to 1.00 (*p* < 0.0001) in six imaging findings, which are shown in Table 3.

**Table 3:** Interobserver agreement for the assessment of findings and visualization comparison between sequences

			**Visual score**				
**Findings (n)**	**Sequences**	**Investigator**	**Mean ± SD**	**Median**	**ICC value**	**Sequences compared**	***p-*** **value**
Patchy increased intensity (69)	UTE	Reader 1	3.12 ± 0.65	3.00	0.83	UTE vs. T2-FSE	< 0.001
		Reader 2	3.00 ± 0.64	3.00			
	T2-FSE	Reader 1	2.09 ± 0.78	2.00	0.79	UTE vs. T1-GRE	< 0.001
		Reader 2	1.86 ± 0.77	2.00			
	T1-GRE	Reader 1	1.84 ± 1.02	2.00	0.87	T2-FSE vs. T1-GRE	0.043
		Reader 2	1.59 ± 1.08	2.00			
Atelectasis/consolidation (15)	UTE	Reader 1	3.60 ± 0.51	4.00	0.87	UTE vs. T2-FSE	0.02
		Reader 2	3.53 ± 0.52	4.00			
	T2-FSE	Reader 1	2.93 ± 0.70	3.00	0.85	UTE vs. T1-GRE	0.035
		Reader 2	2.73 ± 0.88	3.00			
	T1-GRE	Reader 1	3.13 ± 0.74	3.00	0.87	T2-FSE vs. T1-GRE	0.38
		Reader 2	2.93 ± 0.96	3.00			
Nodule/mass (3)	UTE	Reader 1	3.33 ± 0.58	3.00	1	UTE vs. T2-FSE	0.18
		Reader 2	3.33 ± 0.58	3.00			
	T2-FSE	Reader 1	2.33 ± 0.58	2.00	1	UTE vs. T1-GRE	0.1
		Reader 2	2.33 ± 0.58	2.00			
	T1-GRE	Reader 1	0.67 ± 0.58	1.00	1	T2-FSE vs. T1-GRE	0.1
		Reader 2	0.67 ± 0.58	1.00			
Bulla/cyst (4)	UTE	Reader 1	3.75 ± 0.50	4.00	1	UTE vs. T2-FSE	0.06
		Reader 2	3.75 ± 0.50	4.00			
	T2-FSE	Reader 1	1.00 ± 1.41	0.50	0.91	UTE vs. T1-GRE	0.66
		Reader 2	0.75 ± 0.96	0.50			
	T1-GRE	Reader 1	1.50 ± 1.00	2.00	1	T2-FSE vs. T1-GRE	0.41
		Reader 2	1.50 ± 1.00	2.00			
Interstitial lung disease/pulmonary fibrosis (26)	UTE	Reader 1	3.27 ± 0.67	3.00	0.77	UTE vs. T2-FSE	0.83
		Reader 2	3.08 ± 0.63	3.00			
	T2-FSE	Reader 1	3.27 ± 0.78	3.00	0.75	UTE vs. T1-GRE	0.002
		Reader 2	3.04 ± 0.77	3.00			
	T1-GRE	Reader 1	2.38 ± 0.85	2.00	0.83	T2-FSE vs. T1-GRE	0.001
		Reader 2	2.19 ± 0.90	2.00			
Pleural effusion/thickening (4)	UTE	Reader 1	1.50 ± 1.29	1.50	0.92	UTE vs. T2-FSE	0.07
		Reader 2	1.25 ± 1.26	1.00			
	T2-FSE	Reader 1	4.00 ± 0.00	4.00	1	UTE vs. T1-GRE	0.32
		Reader 2	4.00 ± 0.00	4.00			
	T1-GRE	Reader 1	2.00 ± 1.83	2.00	0.96	T2-FSE vs. T1-GRE	0.11
		Reader 2	1.75 ± 1.50	2.00			

The visualization of the Patchy increased intensity was superior in UTE in comparison to T2-FSE (*p* < 0.001) and T1-GRE (*p* < 0.001), and the T2-FSE is better than T1-GRE(*p* = 0.043). In the visualization of atelectasis/consolidation, UTE scored higher than T2-FSE (*p* = 0.02) and T1-GRE (*p* = 0.035), while the difference between T2 and T1 sequences was not statistically significant (*p* = 0.38). Visualization scores of interstitial lung disease/pulmonary fibrosis between UTE and T2-FSE were similar and better than that of T1-GRE (UTE vs. T2-FSE, *p* = 0.83; UTE vs. T1-GRE, *p* = 0.002; T2-FSE vs. T1-GRE, *p* = 0.001). As for the pleural effusion/thickening, T2-FSE (Mean ± SD, 4 ± 0) performed better than the UTE (Mean ± SD, 1.5 ± 1.12) and T1-GRE (Mean ± SD, 2.0 ± 1.58) sequences, although there were no statistically significant differences (UTE vs. T2-FSE, *p* = 0.07; UTE vs. T1-GRE, *p* = 0.32; T2-FSE vs. T1-GRE, *p* = 0.11). In a concrete radiographic score of UTE in nodule/mass and bulla/cyst were higher than the other two sequences, however, no statistically significant differences between UTE with other sequences were found (nodule/mass: UTE vs. T2-FSE, *p* = 0.18; UTE vs. T1-GRE, *p* = 0.1; T2-FSE vs. T1-GRE, *p* = 0.1) (bulla/cyst: UTE vs. T2-FSE, *p* = 0.06; UTE vs. T1-GRE, *p* = 0.66; T2-FSE vs. T1-GRE, *p* = 0.41). The details are shown in Table 3.

## Discussion

During the process of radiological pulmonary inspection for pneumonia, ionizing radiation is a concern for both patients and pediatricians, particularly in the pediatric populations requiring dynamic and longitudinal radiographic follow-up. Due to the lack of ionizing radiation, pulmonary MRI has remained a reliable alternative to CT or X-ray imaging to date. Better lung intrinsic MRI signals visualization, which depends on the used ultrashort or near-zero echo time technique should be anticipated [19]. According to a number of previous studies, UTE MRI has the potential to yield substantial benefits in demonstrating structural bronchial and parenchymal abnormalities [18, 22, 23]. Our work fills a gap in UTE research on neonates by examining the usefulness of pulmonary UTE MRI in young children, including newborns.

This study has successfully demonstrated the feasibility of free-breathing 3D-UTE imaging in the simultaneous pediatric pneumonia detection and higher-quality evaluation of imaging findings in pediatric patients. when compared with frequently used T1-GRE and T2-FSE sequences in pediatric pneumonia, the UTE sequence offers superior image quality for the lung parenchyma, despite the artifact being slightly worse than T1-GRE and equivalent to T2-FSE. UTE can accurately identify abnormalities including patchy increased intensity, atelectasis/consolidation, nodule/mass, bulla/cyst, and interstitial lung disease/pulmonary fibrosis in comparison to routinely used T2-FSE and 3D-T1 GRE sequences. However, when compared to T2-FSE, UTE is inferior in the evaluation of pleural effusion/thickening.

Line with the results of a previous study by Serai et al., who found UTE could provide a higher signal-to-noise ratio from the fast-decaying lung signal in comparison to the conventional spin-echo or gradient-echo sequences [19], the lung parenchyma was consistently imaged better in UTE than in both T2-FSE and T1-GRE sequences. Additionally, we discovered that UTE had somewhat higher artifacts than T2-FSE and T1-GRE, which may contribute to the longer acquisition time and lower thickness. Despite the statistically higher artifacts, there were little or no impact on the clinical diagnosis and assessment of imaging signs. However, it must be acknowledged that one case was rated as limited diagnoses, which may be related to the non-removable treatment equipment worn by the child.

Consolidation and patchy increased intensity are the most common pathological findings in pneumonia. These abnormalities, caused by pneumonia, exhibit higher proton signals than adjacent natural lung parenchyma on MRI sequences. These sequences are characterized by high fluid/interstitium or cellular ratio [8, 24]. The visualization quality of these lesions differed among the three sequences. Specifically, the UTE technique demonstrated significantly higher scores of patchy increased intensity and atelectasis/consolidation in children aged 0–3 years, compared to T2-FSE and T1-GRE. Whereas, a study by Daniel Gräfe et al. on pneumonia in children aged 0–18 years found that the visualization of the pathologies in entities of consolidation was similar between T2-TSE and UTE [18]. The UTE sequence has a distinct advantage in displaying lung parenchyma, allowing for better visualization of areas with increased proton densities compared to spin echo and gradient echo. Additionally, UTE can be comparable to CT in visualizing lesions such as consolidation [25], masses, and nodules [26, 27]. Thin-section UTE MR imaging was found be useful and as effective as CT in evaluating nodule-type lesions. In our study, the advantage of UTE in the demonstration of the nodular sign is evident, however, statistical significance could not be proven due to the small positive sample size.

The UTE imaging technique showed promising results in the visualization of interstitial lung disease/pulmonary fibrosis, which was consistent with the findings of a study conducted by Daniel Gräfe et al. [18] on 56 children. The authors found no significant difference between the UTE and T2-FSE in evaluating the detectability of interstitial patterns and pulmonary fibrosis [18]. It is worth mentioning that both UTE and T2-FSE yielded considerably superior results compared to T1-GRE in detecting interstitial lung disease/pulmonary fibrosis findings. This could be ascribed to the inherent sensitivity of the T2 sequence and UTE in providing high contrast for liquid accumulation [12, 20] accumulation within interstitial tissues and fibrosis, as well their better capability of displaying the normal lung background. However, as the disease progresses and interstitial fluid accumulation is replaced by fibrotic scarring, the signal intensity decreases [12].

No statistically significant difference was found between UTE and the other two sequences in visualizing of pleural effusion/thickening, although there were noticeable differences based on specific scores, indicating that T2-FSE was superior. This can be attributed to the sensitivity of the FSE sequence to the high contrast provided by the liquid accumulation against the black background of normal lung parenchyma. Similarly, although the difference is visible, statistical significance could not be proven in depicting bulla/cyst between sequences. Bulla/cyst were preeminently represented in UTE due to the absence of the signal compared to neighboring lung tissues, which consistent with the results of the study conducted by Daniel Gräfe et al. [18]. Nevertheless, air-filled cysts can be narrowly depicted in T2 and T1 sequences due to the presence of water in the cyst wall, outlining the morphology of the lesion.

Other complicated lesions could still be particularly noteworthy, such as the presence of empyema, necrosis, and abscesses in lung infections, although there was no positive case in our study. Previous studies have shown that MRI has the potential to detect these complications [12, 28], which could have important clinical implications for medication guidance and management of children with pneumonia [17].

In terms of interobserver agreements, significant agreement was observed for image artifacts, pulmonary parenchyma, and pathological imaging findings. This demonstrates that UTE MRI is technically feasible and reproducible for evaluating young children with pneumonia, including newborns.

Smaller and uncooperative younger children and neonates have been rarely included in pulmonary MRI studies due to the safety and anesthesia concerns. Our research not only demonstrated the practicality of UTE sequences in pediatric pneumonia, but also explored and standardized the transportation, heat preservation, anesthesia, and monitoring procedures for children during examinations.

With the improvement of the entire process of lung MRI in younger children and increased experience in anesthesia safety, pediatric pulmonary MRI will become more scientific, standardized, and maneuverable. This will expand the range of pulmonary diseases and complications that can be explored, leading to increased indications for pulmonary MRI and benefiting more pediatric patients in clinical practice.

There are several limitations to this study. First, it included the small sample and single-center recruitment. Second, The lack of clinicopathological data and unclear etiology of pneumonia due to empirical drug use is another limitation. Moreover, children with severe pneumonia or serious complications were not included in the study for safety reasons and guardians' concerns. Last but not least, there were no lung CT examinations available for comparisons, which is considered the “gold standard” imaging modality for pulmonary evaluation. However, the ethical implications of radiation exposure prevented its use.

## Conclusions

In conclusion, UTE MRI proves to be a valuable and potentially applicable technique for evaluating pediatric pneumonia. The UTE sequence provides better or similar visualization of most pathological findings compared to T2-FSE and 3D-T1 GRE in pediatric pneumonia. This technique is particularly suitable for longitudinal and repeated imaging in pediatric patients who require clinical care or research, while minimizing ionizing radiation exposure. Moreover, it holds promise as an inspection modality for various lung diseases in clinical practice. Given the limitations of this study, further multicenter researches are still required to validate and advance the application of UTE MRI in pediatric pulmonary diseases. Subsequent investigations on various pediatric pulmonary diseases with larger sample sizes are desired, including but not limited to tuberculosis, fungal infections, pulmonary tumors, and pulmonary hemorrhage.

## Data Availability

The datasets used and/or analyzed during the current study are available from the corresponding author on reasonable request.

## Abbreviations

BPD: Bronchopulmonary dysplasia
GGO: Ground-glass opacity
3D-UTE: Three-dimensional ultrashort echo time
ICC: Intraclass correlation coefficient
PMA: Postmenstrual age
